# Effectiveness of pharmacological cardioversion of new-onset atrial fibrillation during thoracic surgery operations: a single-centre experience

**DOI:** 10.1186/s13019-023-02236-y

**Published:** 2023-04-07

**Authors:** Dehua WU, Qiongzhen LI, Meiying XU, Jingxiang WU, Jun Yang

**Affiliations:** 1 Department of Anesthesiology, Shanghai Songjiang District Central Hospital, Shanghai, 201600 China; 2 Department of Anesthesiology, Shanghai Chest Hospital, Shanghai Jiao Tong University School of Medicine, Shanghai, 200032 China; 3 Department of Thoracic surgery, Shanghai Chest Hospital, Shanghai Jiao Tong University School of Medicine, Shanghai, 200032 China; 4 Department of Anesthesiology, Songjiang Hospital affiliated to Shanghai Jiao Tong University School of Medicine (Prepare Stage), Shanghai, 201600 China

**Keywords:** Atrial fibrillation, Pharmacological conversion, Electrical cardioversion, Thoracic surgery

## Abstract

**Objective:**

Prophylactic pharmacological conversion agents could reduce the incidence of postoperative atrial fibrillation (AF) in patients undergoing thoracic operations. The current study examined whether the use of pharmacological conversion agents could help to restore sinus rhythm in patients with AF newly developed during thoracic operations.

**Methods:**

Medical records of 18,605 patients from January 1, 2015 to December 31, 2019, at the Shanghai Chest Hospital were reviewed. Patients with non-sinus rhythm prior to the surgery (n = 128) were excluded from data analysis. The final analysis included 18,477 patients (n = 16,292 undergoing lung operations; n = 2,185 undergoing esophageal operations).

**Results:**

Intraoperative AF (defined as AF lasting for at least 5 min) occurred in 646 out of a total of 18,477 subjects (3.49%). Within the 646 subjects, 258 received pharmacological conversion agents during the surgery. sinus rhythm was restored in 20.15% (52/248) of patients treated with pharmacological cardioversion and in 20.87% (81/399) patients not receiving pharmacological intervention. In a subgroup analysis of the 258 patients receiving pharmacological conversion agents, recovery of sinus rhythm was highest in beta-blocker group (35.59%, 21/59 vs. 15.78%, 15/95 in amiodarone group, *p* = 0.008, 5.55%, and 1/18 in amiodarone plus beta-blockers group, *p* = 0.016). The incidence of hypotension was higher in pharmacological conversion (27.5% vs. 9.3% in patients not receiving pharmacological intervention, *p* < 0.001). In subjects not recovering to sinus rhythm during the surgery (n = 513), electrical cardioversion in post-anesthesia care unit (PACU) restored sinus rhythm in > 98% of the cases (155/158 vs. 63/355 in subjects not receiving cardioversion; *p* < 0.001).

**Conclusions:**

Our experience shows that pharmacological conversion, in general, failed to show better treatment effectiveness on intraoperative new-onset AF within period of surgery except for beta-blockers. Patients with AF persisting beyond the surgery could be effectively managed with electrical cardioversion.

Atrial fibrillation (AF) is a common complication in patients undergoing major thoracic operations [1]. Intraoperative incidence is 3.27% and postoperative incidence ranges from 8 to 46% in patients undergoing general thoracic surgery [1]. Perioperative AF is associated with prolonged hospitalization, increased costs, increased morbidity, and even death [2]. A variety of pharmacological conversion agents have been used to manage AF after cardiac surgery and general thoracic surgery. Prophylactic amiodarone could lower incidence of postoperative AF in patients undergoing pulmonary resection [3], as well as in patients undergoing esophagectomy [4]. Similar findings have been reported with other pharmacological conversion agents, including diltiazem [5] and metoprolol [6]. The objective of the current study was to examine whether intraoperative use of pharmacological conversion in new AF patients with general thoracic operations could help to restore sinus rhythm.

## Materials and methods

We retrospectively evaluated 18,605 consecutive patients undergoing elective lung or esophageal operations at the Shanghai Chest Hospital (Shanghai, China) from January 1, 2015 to December 31, 2019. The study was approved by the Institutional Review Board of the Shanghai Chest Hospital (KS1230), and informed consent was waived because of the retrospective nature of this study.

Data were retrieved from electronic anesthesia records. The analysis excluded 128 patients with preoperative non-sinus rhythm. The final analysis included 18,477 patients (n = 16,292 for lung surgery; n = 2,185 for esophageal surgery). Lung surgery included pneumonectomy in 1,034, pulmonary lobectomy in 12,682, pulmonary lobectomy with sleeve resection in 613, and wedge or segment resection in 1,963 subjects. Esophageal surgery included 645 patients with an open left transthoracic approach (Sweet), 1,489 with an open right transthoracic approach (McKeown 968 and Ivor-Lewis 521), and 51 with a closed transthoracic approach (video-assisted thoracoscopic surgery). Lymph node dissection was performed routinely in patients with cancer.

No patient received prophylactic antiarrhythmic treatment. General patient condition was assessed using American Society of Anesthesiology Physical Status immediately prior to surgery.

The types of anesthesia included general anesthesia in 16,809, general anesthesia plus thoracic paravertebral block in 1,560, and general anesthesia plus thoracic epidural block in 108 patients. Anesthesia induction consisted of propofol (1.5 to 2.5 mg/kg; or 2.5 to 4 µg/mL upon target controlled infusion), fentanyl (3 to 5 µg/mg) or sufentanil (0.3 to 0.5 µg/kg), and vecuronium (0.08 to 0.12 mg/kg) or rocuronium (0.6 to 0.9 mg/kg). Anesthesia was maintained with total intravenous anesthesia. Fentanyl (or sufentanil) was supplemented as needed. For paravertebral block (Th 4 to 8), 0.375% ropivacaine (20 mL) was injected. For epidural block, a catheter was inserted at the Th 6 to 7 or the Th 7 to 8 level before anesthesia induction for continuous infusion of 0.25% ropivacaine.

Electrocardiogram, oxygen saturation by pulse oximetry, and end-tidal carbon dioxide were monitored. Blood pressure was monitored non-invasively before anesthesia induction, and invasively afterwards. Central venous pressure was monitored routinely.

AF was identified based on intraoperative anesthesia notes exclusively, and defined as AF episode lasting for at least 5 min, and managed based on the discretion of the anesthetists. The type and dosage of the pharmacological conversion agents (if used during the surgery) are shown in Table 1. Effectiveness of pharmacological conversion agents in the current study was defined as restoration of sinus rhythm at the end of surgery. In some of the patients with AF persisting beyond surgery, biphasic electrical cardioversion was carried out under the influence of anesthesia or sedation in post-anesthesia care unit (PACU). The cardioversion started with 100 J, and repeated with higher energy level of 150 J and then 200 J if necessary (max number: 3). Necessary treatments, such as correcting acid/base and electrolyte disturbances, hypoxia and hypothermia, were implemented prior to electrical conversion.

The intraoperative cardiovascular adverse effects in timing relative to the onset of AF and pharmacological cardioversion were defined as follows: tachycardia (heart rate > 100 beats/min), bradycardia (heart rate < 50 beats/min and/or treated with atropine), hypotension (mean blood pressure < 50 mmHg and/or treated with vasoactive drugs, such as phenylephrine, dopamine, ephedrine and noradrenaline) and severe hypotension (systolic pressure < 60 mmHg or mean blood pressure < 30 mmHg).

**Table 1:** Type and dosage of pharmacological conversion agents

Pharmacological conversion agents	Loading dose	Maintenance dose
Amiodarone	75 to 150 mg over 10 min	0.5 to 1 mg/min
Esmolol	500 mcg/kg over 1 min	0.05 to 0.2 mg/kg/min
Diltiazem	0.25 mg/kg over 2 min	5 to 15 mg/h
Propafenone	0.5 to 1 mg/kg over 5 min	20 to 40 mg/h
Propranolol	0.15 mg/kg over 10 min	-
Digoxin	0.2 to 0.4 mg over 10 min	-
Lidocaine	1.0 to1.5 mg/kg over 5 min	-

The dosage in drug combination differed (typically lower) from that listed above.

Statistical analysis was performed using SPSS 16 software (SPSS Inc, Chicago, Ill). All statistical tests were two-sided. Statistical significance was set at *p* < 0.05. The Student’s *t*-test was used to compare continuous variables in patients receiving pharmacological conversion agents vs. those who did not; and Mann-Whitney *U*-test for continuous variables of abnormal distribution. The χ^2^ test or Fisher exact test, as appropriate, was used for analysis of categorical variables. For analysis of different pharmacological conversion agents, and *p* was adjusted using Bonferroni method for multiple comparisons.

## Results

Intraoperative AF occurred in 646 of 18,477 patients (3.49%). The incidence of intraoperative AF was 3.41% (556/16,292) in lung operations and 4.12% (90/2,185) in esophageal operations (*p* = 0.091). In esophageal operations, the incidence of AF was higher in the left transthoracic approach than in the right transthoracic approach (6.35%, 41/645 vs. 3.18%, 49/1,540; *p* = 0.001).

The following measures did not differ between AF patients receiving pharmacological conversion agents or not: gender, age, lung vs. esophageal surgery, operation complexity, anesthesia type and duration, comorbid conditions, and ASA classification (Table 2).

The timing of onset of AF after operation beginning and the incidence of AF during different periods showed no statistical difference between the 2 groups of receiving pharmacological conversion agents and not receiving pharmacological intervention. Blood pressure and heart rate before anesthesia induction, at 5–10 min prior to the onset of AF and 3–5 min after AF between the 2 groups also did not show either statistically significant or clinically meaningful differences (Table 3).

**Fig. 1:**
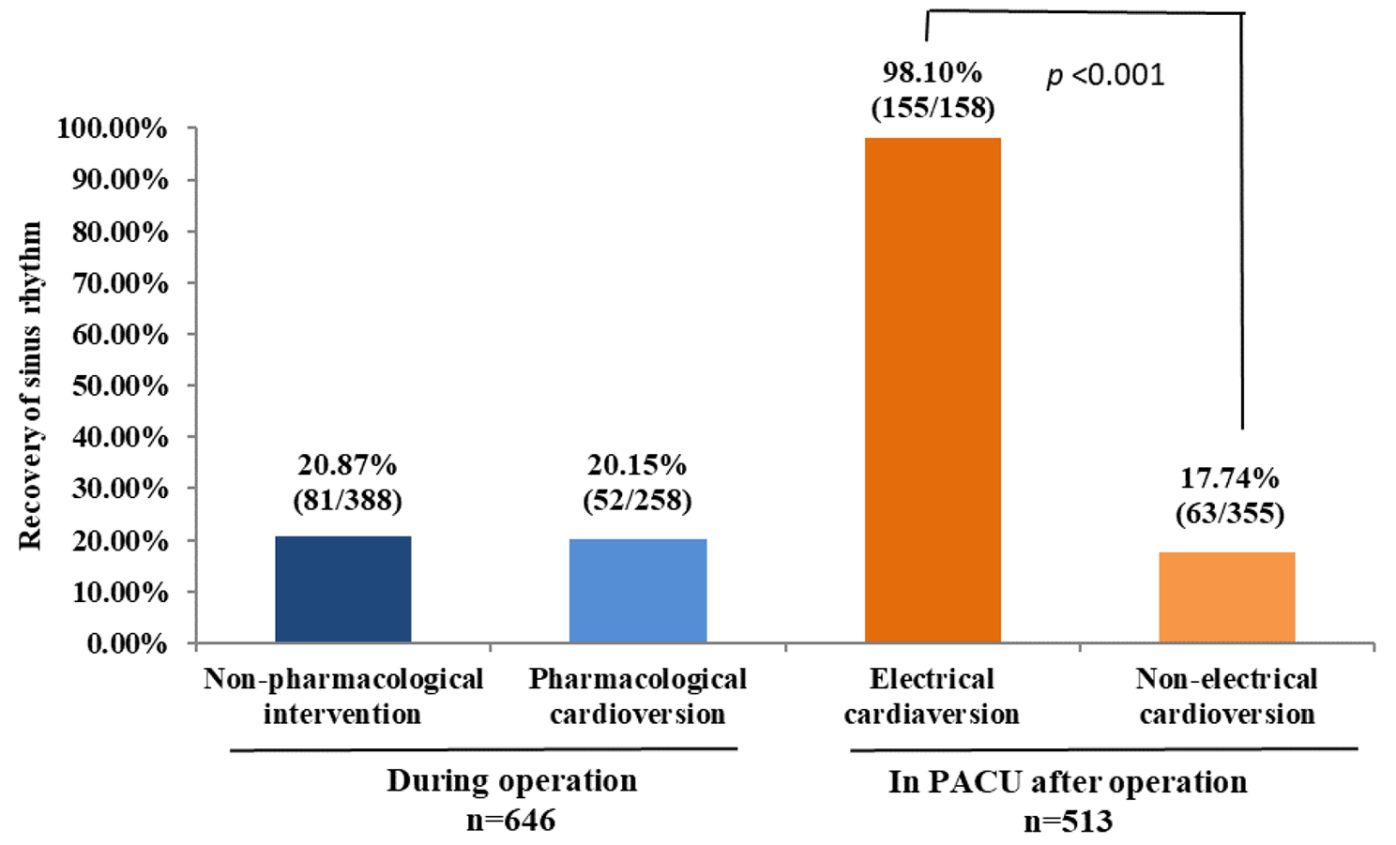
Recovery of sinus rhythm during surgery and in PACU. AF was defined as AF episode lasting at least 5 min, restoration was defined as a conversion into a stable sinus rhythm. Data were analyzed with χ^2^ test

**Fig. 2:**
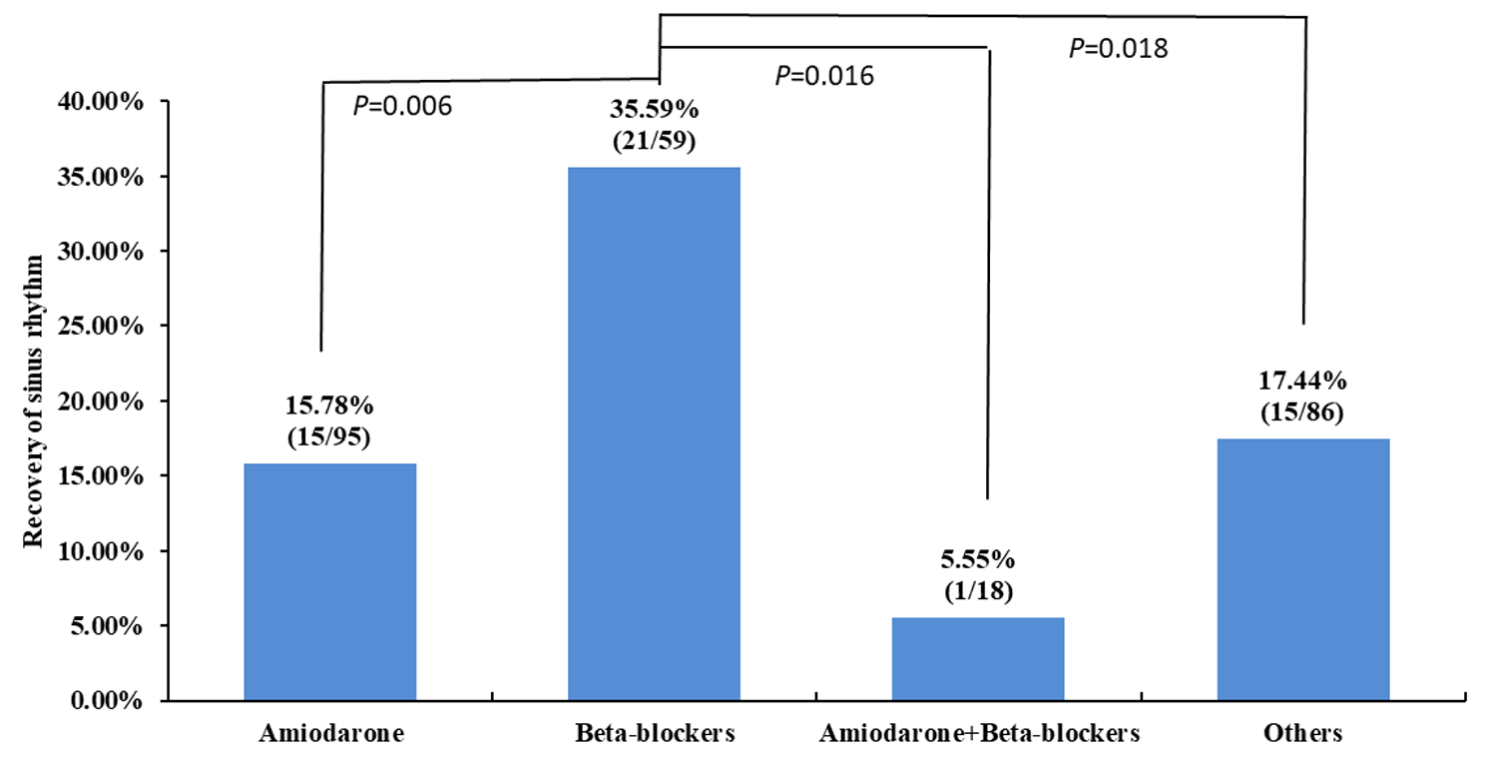
Recovery of sinus rhythm after various pharmacological conversion agents during surgery. The overall analysis was conducted with the χ^2^ test. Pairwise comparison was carried out using Bonferroni method and *p* was adjusted to 0.017 (0.05/3). Amiodarone group: patients receiving amiodarone, and possibly other agents but not beta-blockers; beta-blockers group: patients receiving beta-blockers, and possibly other agents but not amiodarone; amiodarone + beta-blockers group: patients receiving both amiodarone and beta-blockers, and may receive additional agents; other group: patients receiving other pharmacological conversion agents, but not amiodarone or beta-blockers, including propafenone, digoxin, lidocaine, diltiazem, as well as a variety of combination

In patients who developed AF during the surgery, sinus rhythm was restored in 20.15% (52/248) of patients treated with pharmacological cardioversion and in 20.87% (81/399) patients not receiving pharmacological intervention (*p* = 0.824, Fig. 1). In a subgroup analysis of the 258 patients receiving pharmacological conversion, the number of the patients receiving each drug was 95(36.8%) for amiodarone, 59 (22.9%) for beta-blockers, 18 (7.0%) for amiodarone plus beta-blockers, 86 (33.3%) for others (diltiazem 20, propafenone 21, digoxin 27, lidocaine 18), recovery of sinus rhythm was highest in beta-blocker group (35.59%, 21/59 vs. 15.78%, 15/95 in amiodarone group, *p* = 0.006, and 5.55%, 1/18 in amiodarone plus beta-blockers group, *p* = 0.016) (Fig. 2).

Measures that potentially reflect the cardiovascular adverse events of pharmacological conversion agents are summarized in Table 4. Tachycardia during 5–30 min after AF (heart rate > 100 beats/min) was lower in the pharmacological conversion group (0% vs. 6.7% in patients not receiving pharmacological intervention, *p* < 0.001). The incidence of hypotension was higher in pharmacological conversion group (27.5% vs. 9.3% in patients not receiving pharmacological intervention, *p* < 0.001). In pharmacological conversion group, 7 of 258 (2.7%) patients developed severe hypotension with systolic pressure < 60 mmHg during 5–30 min after AF (after receiving pharmacological conversion).

AF persisted beyond the surgery in 513 patients: 158 AF patients were treated with electrical cardioversion in PACU, 155 (98.1%) restored to sinus rhythm; the remaining 355 patients did not receive cardioversion or any pharmacological intervention in PACU, sinus rhythm recovered spontaneously in 17.74% (*p* < 0.001) (Fig. 1).

**Table 2:** Demographic, General Information and Statistical Analysis

Characteristics	Non-pharmacological intervention (n = 388)	Pharmacological conversion (n = 258)	*p*-value
Age, years	60.73 ± 8.9	60.22 ± 8.0	0.458
Gender			0.645
Male	332 (85.6)	225 (87.2)	
Female	56 (14.4)	33 (12.8)	
Operation site and complexity			0.827
Lungs	333 (85.8)	223 (86.4)	0.920
Pulmonary wedge or segment resection	4 (1.2)	2 (0.9)	
Pulmonary lobectomy	268 (80.5)	185 (83.0)	
Pulmonary lobectomy with sleeve resection	25 (7.5)	15 (6.7)	
Pneumonectomy	36 (10.8)	21 (9.4)	
Esophagus	55 (14.2)	35 (13.6)	0.964
Sweet operation	18 (32.7)	12 (34.3)	
Mckeown operation	22 (40.0)	13 (37.1)	
Ivor-Lewis operation	15 (27.3)	10 (28.6)	
Approach			0.124
Video-assisted thorascopic surgery	81 (20.9)	41 (15.9)	
Open operation	307 (79.1)	217 (84.1)	
Laterality of operation			0.066
Right	236 (60.8)	176 (68.2)	
Left	152 (39.2)	82 (31.8)	
Anesthesia type			0.798
General	369 (95.1)	249 (96.5)	
General + paravertebral block	17 (4.4)	8 (3.1)	
General + epidural block	2 (0.5)	1 (0.4)	
Duration of anesthesia, min	216.4 ± 61.5	221.3 ± 45.6	0.268
Co-existing baseline diseases			
Hypertension			0.544
Yes	32 (8.3)	17 (6.6)	
No	356 (91.7)	241 (93.4)	
Diabetes mellitus			0.523
Yes	28 (7.2)	15 (5.8)	
No	360 (92.8)	243 (94.2)	
Coronary artery disease			0.747
Yes	7 (1.8)	3 (1.2)	
No	381 (98.2)	255 (98.8)	
Respiratory deficiency			0.902
Yes	7 (1.8)	5 (1.9)	
No	381 (98.2)	253 (98.1)	
Obesity			0.925
Yes	13 (3.4)	9 (3.5)	
No	375 (96.6)	249 (96.5)	
ASA classification			0.339
I	23 (5.9)	9 (3.5)	
II	302 (77.8)	215 (83.3)	
III	61 (15.7)	33 (12.8)	
IV	1 (0.3)	1 (0.4)	
V	1 (0.3)	0 (0)	

Continuous variables are shown as mean ± standard deviation; categorical variables as number (%). AF = atrial fibrillation; ASA = American Society of Anesthesiology; Respiratory deficiency: forced expiratory volume in 1 s (FEV1%) < 50%; Obesity was defined as: body mass index ≥ 30 kg/m^2^.

**Table 3:** Timing of AF occurrence, pharmacological conversion agents use, and timing from antiarrhythmic drug administration to pharmacological cardioversion and BP and HR at the time of baseline, before and after AF occurrence

	Non-pharmacological intervention (n = 388)	Pharmacological conversion (n = 258)	*p*-value
Timing of AF occurrence after operation beginning [median (IQR), min]	75 (50)	85 (45)	0.139
Different periods of AF occurrence during operations			0.095
Entering thoracic cavity (n, %)	10 (2.6)	4 (1.6)	
Targeted lung or esophagus resection (n, %)	91 (23.5)	83 (32.2)	
Lymph node dissection (n, %)	232 (59.8)	141 (54.7)	
Chest closure (n, %)	55 (14.2)	31 (12.0)	
Timing of receiving pharmacological conversion agents [median (IQR), min]	-	5 (3)	-
Timing from antiarrhythmic drug administration to pharmacological cardioversion [median (IQR), min]	-	66 (67.5)	-
SBP			
Before anesthesia (mmHg)	136.67 ± 21.23	134.48 ± 25.71	0.238
5–10 min before AF (mmHg)	117.81 ± 16.97	120.31 ± 17.38	0.185
3–5 min after AF (mmHg)	117.37 ± 16.67	114.89 ± 20.31	0.232
BDP			
Before anesthesia (mmHg)	76.76 ± 11.56	75.89 ± 13.47	0.381
5–10 min before AF (mmHg)	64.00 ± 9.51	65.53 ± 9.97	0.152
3–5 min after AF (mmHg)	65.86 ± 10.83	63.96 ± 11.84	0.127
HR			
Before anesthesia (beats/min)	75.78 ± 10.51	75.59 ± 11.66	0.829
5–10 min before AF (beats/min)	76.55 ± 16.20	74.79 ± 11.97	0.247
3–5 min after AF (beats/min)	82.76 ± 20.26	86.43 ± 23.64	0.131

Continuous variables are shown as mean mean ± standard deviation or median (IQR). AF: atrial fibrillation; DBP: diastolic blood pressure; HR: heart rate; IQR: Interquartile range; SBP: systolic blood pressure

**Table 4:** Intraoperative cardiovascular adverse events in timing relative to the onset of AF and pharmacological cardioversion

	5–10 min before AF				5-30 min after AF		
	Non-pharmacological intervention	Pharmacological conversion	P-value		Non-pharmacological intervention	Pharmacological conversion *	P-value
Tachycardia (n, %)	8 (2.1)	5 (1.9)	0.577		26 (6.7)	0 (0)	< 0.001
Bradycardia (n, %)	0 (0)	0 (0)	-		0 (0)	4 (1.6)	0.025
Hypotension (n, %)	0 (0)	2 (0.8)	0.159		36 (9.3)	71 (27.5)	< 0.001
Mild and moderate	0 (0)	2 (0.8)	0.159		36 (9.3)	64 (24.8)	< 0.001
Severe	0 (0)	0 (0)	-		0(0)	7 (2.7)	0.002

Data are presented as n (%) of patients. AF: atrial fibrillation; Bradycardia: heart rate < 50 beats/min (bpm); Hypotension: mean blood pressure < 50mmHg; Severe hypotension: systolic pressure < 60 mmHg or mean blood pressure < 30 mmHg and need to treat urgently; Tachycardia: heart rate > 100 bpm. * Adverse events after pharmacological cardioversion agents.

### Comment

The rate of restoration to sinus rhythm within period of surgery did not differ between those receiving vs. not receiving antiarrhythmic agents (20.87% vs. 20.15%) in general. Comparing to other antiarrhythmic agents, beta-blockers showed better treatment effectiveness for new-onset atrial fibrillation. Electrical cardioversion in PACU could restore sinus rhythm in > 98% of the cases.

Different mechanisms were responsible for new-onset AF occurrence during vs. after the operation [7]. Decreased cardiopulmonary reserve and systemic inflammatory response to the surgery, enhanced activity of the sympathetic nervous system were important contributing factors to postoperative AF [8]. In contrast, the major factor that triggers intraoperative AF was mechanical stimulation, particularly to cardiac plexus, and near the pulmonary veins [7]. In the current study, the incidence of intraoperative AF was likely higher in patients receiving esophageal vs. lung operation. Such a finding probably reflects closer proximity of esophagus to the left atrium, richer innervation of esophagus by both sympathetic and parasympathetic fibers and thus greater mechanical stimulation to those areas. We also noticed higher incidence of intraoperative AF in esophageal surgery with left approach, mostly likely due to greater stimulation of cardiac plexus that is located in the left thoracic cavity.

The mechanisms underlying new AF during vs. after thoracic surgery differ significantly. Most notably, new-onset AF during surgery is primarily due to mechanical stimulation of atria and pulmonary veins and the autonomic nerves, and thus is less likely to respond to drug treatments. In contrast, prophylactic use of antiarrhythmic drugs (e.g., amiodarone, diltiazem, and metoprolol) has been shown to be effective for postoperative AF [4–6].

However, patients treated in beta-blockers for new AF showed higher recovery of sinus rhythm comparing with others. These might be explained by the effect of beta-blocker’s appropriate rate control, inhibiting heart sympathetic adrenergic tone and balancing sympathetic and vagal tone [9]. Beta-blockers are most effective when adrenergic tone is high [10]. We speculate that the apparent effectiveness of beta-blockers vs. amiodarone reflects the true pharmacological effects of beta-blockers as well as the higher heart rate. But the efficacy of beta-blocker on AF to restore sinus rhythm was still low with less than 40% in this study.

In the current study, we noticed a variety of important albeit infrequent side effects of pharmacological cardioversion agents, including bradycardia and hypotension. Many of the currently available drug therapies for AF have limitations [11]. Intraoperative new AF is more likely to produce tachycardia, however hypotension is more likely to be caused after using pharmacological cardioversion agents in our current study. The rate of intraoperative hypotension in the non-pharmacological intervention group in the current study (9.3%) was comparable to that reported by Hou and colleagues in 11 patients who developed new-onset AF undergoing esophageal resection (9.1%) [12]. This finding adds to the robustness of higher rate of intraoperative hypotension in the pharmacological conversion group (24.8% for mild and moderate hypotension, 2.7% for severe hypotension) vs. the non-pharmacological conversion group. Lower blood pressure with pharmacological cardioversion is mostly due to the vasodilating effects rather than negative inotropic effects [13, 14]. So we speculate that using pharmacological cardioversion drugs for new AF during operation could affect hemodynamic stability. Monoe et al. [15] reported that another most common side effect was bradycardia because of longer duration of amiodarone use.

Electrical cardioversion is widely used to manage AF [16]. In the current study, 98.1% of new AF were restored to sinus rhythm by electrical cardioversion (vs. 17.74% in those not receiving electrical cardioversion). Notably, electrical cardioversion is not suitable or inconvenient during operations because of surgical manipulation. In PACU, electrical cardioversion could be readily performed since the patients have not recovered from anesthesia and also due to tracheal intubation. Nuotio et al. [17] reported that cardioversion at 12 h or later from AF onset is associated with higher risk of thromboembolic complications (1.1%). Anticoagulant therapy is not needed at this point because the duration of AF is less than 12 h in the current study.

The results of the current study suggested that using pharmacological conversion drugs during thoracic surgery does not help to restore sinus rhythm. In addition, using pharmacological conversion could produce hemodynamic instability. However, a subgroup analysis of the patients receiving pharmacological conversion showed higher recovery of sinus rhythm in patients receiving beta-blockers than other agents. Since beta-blockers are the first-line drugs for AF in general recommended [18], we speculate that beta-blockers could be used in patients with high sympathetic adrenergic tone during operations. Further studies are required to test this hypothesis.

### Limitations

This is a retrospective analysis. Potential biases include non-randomization of the use of pharmacological conversion vs. non-pharmacological intervention: the choice is based on physician discretion and experience. The pharmacological conversion agents also varied considerably, including amiodarone, beta-blockers, propafenone, digoxin, lidocaine, diltiazem. Also, the endpoint of observation in the current study was the completion of the surgery. Therefore, lack of effects of these agents on intraoperative AF in the current study does not necessarily exclude its clinical benefit beyond the surgery.

The observational nature of the current study could be viewed as an advantage as well. Ideally, the effects of pharmacological conversion should be observed in patients not receiving electroversion in PACU. However, such a practice (no electroversion despite of AF persistence after surgery) is not in the best interest of the patients in our opinion.

## Conclusion

Pharmacological conversion generally failed to show better treatment effectiveness on intraoperative new-onset AF within period of surgery except for beta-blockers. Patients with AF persisting beyond the surgery could be effectively managed with electrical cardioversion.

## Data Availability

Not applicable.
